# Supplementary material for: Information Retrieval and Awareness about Evidence-Based Dentistry among Dental Undergraduate Students—A Comparative Study between Students from Malaysia and Finland
Source: Dent J (Basel). 2020 Sep 3;8(3):103. doi: 10.3390/dj8030103 (PMC7558687; doi:10.3390/dj8030103)
Supplement: Supplementary file 1 [file dentistry-08-00103-s001.pdf]

The aim of this survey is to investigate dental students and graduates knowledge of evidence-based dentistry, and to find out what information sources they use in their dental work.

Background characteristics

1. Year of study: Year 3 ☐ Year 4 ☐ Year 5 ☐

2. Gender: ☐ Male ☐ Female

Evidence-based dentistry

3. Assessing level of evidence.

Please rate the following alternatives for the level of evidence they exhibit on a scale of 1 – 4, with most reliable information as 1 and least reliable as 4.

- ☐ Cohort or longitudinal studies
- ☐ Randomized controlled trials or observational studies with a significant effect
- ☐ Case-control studies
- ☐ Systematic reviews and meta-analyses of randomized controlled trials
- ☐ I don't know

4. What is meta-analysis?

- ☐ A statistical method in which results from several studies have been combined and analyzed using quantitative methods
- ☐ Tool to evaluate the quality of systematic reviews
- ☐ Summary of studies with clear clinical significance
- ☐ I don't know

5. What is PICO?

- ☐ Abbreviation to describe the elements of good clinical questions
- ☐ Abbreviation of a collaboration group, which has defined criteria for treatment guidelines
- ☐ Abbreviation for evidence-based treatment phases in dental clinic
- ☐ I don't know

Information retrieval as an undergraduate dental student

6. Rate your personal skills in searching for scientific information

- ☐ Excellent
- ☐ Good
- ☐ Satisfactory
- ☐ Mediocre
- ☐ Poor

7. How often have you used the following information sources as an undergraduate student in the last six months?

	Daily	Weekly	Less than weekly	Never
Dental textbooks				
Other textbooks				
Colleagues				
Current clinical practice guidelines by Oral health division Of Ministry of Health				
Other guideline, Please name				
National Dental Journals/ Journals of local Universities				
Evidence Based Dentistry journals				
Other dental journals				
Cochrane library				
Medline / PubMed article database				
Personal lecture notes				
Advertisements				
Other sources, Please name				

8. Do you easily find the needed scientific information from the **Internet**?

☐ Yes ☐ No ☐ I don't know

9. Have you used any one of the following online databases?

	Daily	Weekly	Less than	Never
Pharmacological database				
Pharmaceutical safety database				
Oral Health –database				
Guidelines for medical and dental practitioners				
Patient instructions				
Other				

Others, Specify____

10. How often have you used the following information sources **outside the college working hours** during the past 6 months?

	Daily	Weekly	Less than weekly	Never
Dental textbooks				
Other textbooks				
Colleagues				
Current clinical practice guidelines by Oral health division Of Ministry of Health				
Other guideline, Please name				
National Dental Journals/ Journals of local Universities				
Evidence Based Dentistry journals				
Other dental journals				
Cochrane library				
Medline / PubMed article database				
Personal lecture notes				
Advertisements				
Other sources, Please name				

11. How often have you used the following information sources **at the polyclinics in the college** during the past 6 months?

	Daily	Weekly	Less than weekly	Never
Dental textbooks				
Other textbooks				
Colleagues				
Current clinical practice guidelines by Oral health division Of Ministry of Health				

Other guideline, Please name

National Dental Journals/ Journals of local Universities				
Evidence Based Dentistry journals				
Other dental journals				
Cochrane library				
Medline / PubMed article database				
Personal lecture notes				
Advertisements				
Other sources, Please name				

12. Which are your primary information sources from the list above? Why do you use these?

____

____

____

13. In which dental specialist areas have you searched for scientific information from different information sources? Choose **three** specialist areas you have most frequently searched for information.

- ☐ Oral surgery
- ☐ Implantology
- ☐ Endodontics
- ☐ Prosthodontics
- ☐ Cariology
- ☐ Periodontology
- ☐ Oral radiology
- ☐ Orthodontics
- ☐ Pedodontics
- ☐ Oral Medicine and Oral Pathology
- ☐ Other, which? ______________

14. Regarding evidence based dentistry, anything else you would want to add____________________

____

Thank you for your contribution!
